# Developing the national community health assistant strategy in Zambia: a policy analysis

**DOI:** 10.1186/1478-4505-11-24

**Published:** 2013-07-20

**Authors:** Joseph Mumba Zulu, John Kinsman, Charles Michelo, Anna-Karin Hurtig

**Affiliations:** 1 Department of Public Health, School of Medicine, University of Zambia, P.O. Box 50110, Lusaka, Zambia; 2 Umeå International School of Public Health (UISPH), Umeå University, Umeå SE 90185, Sweden

**Keywords:** Human resources, National community health assistant strategy, Policy analysis, Zambia

## Abstract

**Background:**

In 2010, the Ministry of Health in Zambia developed the National Community Health Assistant strategy, aiming to integrate community health workers (CHWs) into national health plans by creating a new group of workers, called community health assistants (CHAs). The aim of the paper is to analyse the CHA policy development process and the factors that influenced its evolution and content. A policy analysis approach was used to analyse the policy reform process.

**Methodology:**

Data were gathered through review of documents, participant observation and key informant interviews with CHA strategic team members in Lusaka district, and senior officials at the district level in Kapiri Mposhi district where some CHAs have been deployed.

**Results:**

The strategy was developed in order to address the human resources for health shortage and the challenges facing the community-based health workforce in Zambia. However, some actors within the strategic team were more influential than others in informing the policy agenda, determining the process, and shaping the content. These actors negotiated with professional/statutory bodies and health unions on the need to develop the new cadre which resulted in compromises that enabled the policy process to move forward. International agencies also indirectly influenced the course as well as the content of the strategy. Some actors classified the process as both insufficiently consultative and rushed. Due to limited consultation, it was suggested that the policy content did not adequately address key policy content issues such as management of staff attrition, general professional development, and progression matters. Analysis of the process also showed that the strategy might create a new group of workers whose mandate is unclear to the existing group of health workers.

**Conclusions:**

This paper highlights the complex nature of policy-making processes for integrating CHWs into the health system. It reiterates the need for recognising the fact that actors’ power or position in the political hierarchy may, more than their knowledge and understanding of the issue, play a disproportionate role in shaping the process as well as content of health policy reform.

## Background

Health workforce deficits have posed a critical constraint to health system performance in many low and middle income countries (LMIC) [1]. The inability of countries to train, retain and distribute health workers threatens individuals, communities and the attainment of all the health-related Millennium Development Goals [2]. The World Health Organization (WHO) has estimated that 57 countries face critical health worker shortages, of which 36 (63%) are in sub-Saharan Africa [3]. This crisis has been exacerbated by the stress which the demand for HIV-care places on already overstretched health systems [4-6].

Strategies for resolving the human resource crisis include promoting collaboration among multiple sectors and bringing together stakeholders who may have complementary roles. For example, in 2009, the Global Health Workforce Alliance (GHWA) – an innovative partnership whose aim is to identify and coordinate solutions to the health workforce crisis – developed the Country Coordination and Facilitation (CCF) approach. The CCF approach requires establishing and supporting the necessary governance structures for inter-sectoral coordination and collaboration in order to plan, implement and monitor health workforce development and retention at the country level. It also helps priority countries to ensure that sustainable, motivated and skilled health workers are available to meet healthcare needs and work with partners so as to ensure that funding and technical expertise are available for programmes [2].

Apart from promoting staff retention and retraining, other strategies for addressing the human resources crisis include task shifting, which involves reviewing and delegating as many tasks as possible away from doctors, nurses and pharmacists to non-clinical staff, thereby enabling clinical staff to concentrate on their specific areas of expertise [1,3,7,8]. Task shifting can also involve the creation of new cadres to extend workforce capacity [9].

Several countries have begun to formalize the practice of task shifting for health services as one way of addressing the human resources crisis [9,10]. It is in the context of task shifting that the concept of using community health workers (CHWs) to render certain basic health services to their communities has regained currency [1]. CHWs are members of the communities where they work, selected by their communities, and answerable to the communities for their activities. Although they may be supported by the health system as they perform a wide range of tasks that can be preventive, curative and developmental in nature, they have less training than professional workers [11].

Recently, some countries have attempted to formalize CHWs. For example, in Malawi and Uganda, the basic care package for people living with HIV has been designed to be delivered by non-specialist doctors or nurses supported by CHWs [6,9]. Similarly, Ethiopia has implemented a plan to hire Health Extension Workers under the Health Extension Programme (HEP). The HEP aims to “*reduce geographical barriers to care. As a result between 2004 and 2008, the percentage of births with a skilled attendant doubled, and the percentage of women receiving antenatal care and of infants fully immunized increased by over 50%*” [12], p. 88].

There is a good body of evidence showing that CHWs can significantly add to the efforts of improving the health of the population [13,14]. Studies conducted on some HIV programmes that make use of CHW services have shown that CHWs contribute positively to HIV interventions [9,15]. Further, a global systematic review commissioned by GHWA on CHW interventions, as well as eight in-depth country case studies in sub-Saharan Africa (Ethiopia, Mozambique and Uganda), Asia (Bangladesh, Pakistan and Thailand) and Latin America (Brazil and Haiti) on wide-scale use of CHWs also demonstrated positive results [2,9,13]. It was observed that CHWs provide a critical link between their communities and the health and social services system. Following this study, GHWA convened a global consultation of programme managers, policy makers and experts in 2010 to develop a broad agreement on key messages on the role of CHWs in health service delivery. One key message was that countries should consider integrating CHWs fully into national human resources for health (HRH) plans and health systems [13,16]. Given this background, the strategy developed in Zambia for CHWs represented a fundamental shift in community-based health workforce policy.

### Community-based health workforce in Zambia

Like other LMICs, Zambia, with a population of 13.5 million people is facing a chronic shortage of human resources for health. This challenge has been worsened by the extra demands brought about by the high burden of HIV infection, malaria, tuberculosis and other health problems. HIV prevalence in the adult Zambian population stands at 13.5%; average life expectancy in Zambia is a mere 48 years; neonatal mortality is 35 per 1,000 live births; while infant and under-five mortality are also high, at 86 and 141 per 1,000 live births, respectively [17]. The proportion of vacant posts has been estimated at 42% in rural health centres, 22% in urban health centres, and 41% in hospitals. Vacancies for nurses, the most numerous clinical care category in Zambia, stand at 55%, as compared with 63% and 64% of clinical officer and doctor positions, respectively. Distribution of the health workforce is also highly uneven, with rural areas facing a more significant shortage than urban areas [17].

There are six levels of care in the public sector and corresponding facilities, including outreach services, health posts, health centres, and level-1 district, level-2 provincial and level-3 central hospitals [18]. The health post is the lowest level health facility, while nurses are the lowest category of formal health workers. Recent estimates suggest that Zambia has about half the health workforce that it needs, with fewer than 646 doctors and 6,096 nurses [17,19].

Because of this shortage, about 23,500 CHWs have been active providing outreach services during the past 10 years, although this has been going on for a longer period in Kalabo district of Western Province, where a CHW pilot programme was officially launched in 1983 [19,20]. In Zambia, CHWs are conceived as “*members of communities who work either for pay or as volunteers in association with the local health care system and usually share ethnicity, language, socio-economic status and life experiences with the community members they serve. They have many titles, including home based care givers (often work with faith-based programmes), health promoters, community health advisors, lay health advocates, community health representatives, and peer health educators*” [19], p. 8].

Community health workers either work for health care facilities or NGOs. In both instances they help in providing basic health services in areas with geographical access challenges and where there is a shortage of professional health workers. For example, CHWs have been able to manage malaria fevers by correctly interpreting results and appropriately prescribing antimalarials [21]. Other activities include promoting proper food production, basic sanitation, and detecting risk groups for the prevention of common illness [21]. In HIV prevention, treatment and care programs, CHWs organize support groups and perform home visits for patients who miss their appointments, clearly greatly contributing to the supply of health service and in particular to the rapid expansion of antiretroviral drug delivery [22]. Despite this, there has been no comprehensive community health policy and strategy to guide the operations of this workforce, nor has there been any comprehensive structure to regulate and monitor the services they offer. The mandate to regulate their services is often left to individual institutions or organisations engaging them.

With increasing external pressures and funding, in particular for HIV/AIDS control, CHWs have been required to perform new tasks with different training and incentive packages, and this has ultimately caused further fragmentation in their operations [23]. Additional challenges that CHW programs face in Zambia include high turnover, low motivation, inadequate supervision, insufficient compensation or incentives, and low recognition by qualified health care providers, all of which limit their ability to effectively contribute towards primary health care [17,19].

### The national community health assistant strategy

To try and address some of the challenges that CHWs face in health service delivery, the Zambian Ministry of Health (MoH) developed the National Community Health Assistant Strategy in 2010 [19]. The purpose of this strategy is to formalize and standardize the role of CHWs in the health system by creating a new group of workers called community heath assistants (CHAs). Compared to CHWs, whose training is short and not standardised, CHAs are expected to undergo a year’s standardised training programme. Furthermore, in contrast to CHWs who work as volunteers, CHAs will be put on the government payroll and registered with a regulatory body. The CHAs are expected to work below nurses, and they deliver health services on a task-shifting basis (i.e., from nurses to CHAs). Their tasks and mode of supervision are clearly specified in the strategy. The Neighbourhood Health Committees (committees comprising leaders in the community) and the District Health Management Team are responsible for selecting CHAs from the communities in the district. Implementation of the strategy started in June 2011 with a pilot phase lasting until 2013. During this first phase, 307 CHAs were trained. If successful, the strategy will be rolled out to all districts in Zambia in four phases, from 2013 to 2017. About 5,200 CHAs will be trained by 2017 [19].

Since the CHA strategy was the first policy framework aimed at integrating CHWs into the health system in Zambia and since it was only launched in 2010, little analysis has been performed, to date, on how power dynamics among various actors and contextual factors shaped the process of producing the strategy and its content. Current studies on the CHA strategy in Zambia have focused more on considerations relevant to selecting and compensating workers at the community level [24].

This paper, therefore, seeks to fill this knowledge gap by addressing the following questions. First, which actors contributed towards the development of the CHA strategy, and what were their roles? Second, how did the process of agenda setting and developing the CHA strategy evolve? Third, to what extent did actors’ differential power and other contextual factors shape the development and content of the policy? These questions are not only relevant to the MoH in Zambia but also to organizations and other countries that intend to introduce health reforms aimed at strengthening community participation in health care services.

### Conceptual framework

The “policy making process” refers to the way in which policies are initiated, developed or formulated, negotiated, communicated, implemented, and evaluated. This process often occurs in stages, which include problem identification and issue recognition (i.e., focusing on getting issues onto the policy agenda), and policy formulation, which includes actor involvement in formulating policy, how policies are arrived at, agreed upon, and how they are communicated. The other stages are policy implementation as well as policy monitoring and evaluation [25].

The policy analysis framework of Walt and Gilson will guide analysis of the findings [26]. This framework recognizes that the health policy process involves four elements, namely (i) the context within which the policy is formulated and executed, (ii) the actors involved in policy making, (iii) the steps associated with the development process, and (iv) the policy content. In this paper, the analysis approach is guided by the assumption that actors, such as the CHA strategic team, directly influence the course that any policy process will take, as well as the final content of the policy through their actions and values [27]. Further, we assume that actors’ views are influenced by the context in which they live and work, while their role in policy development is dependent on their power [25,26].

## Methodology

### The study site

**Figure 1:**
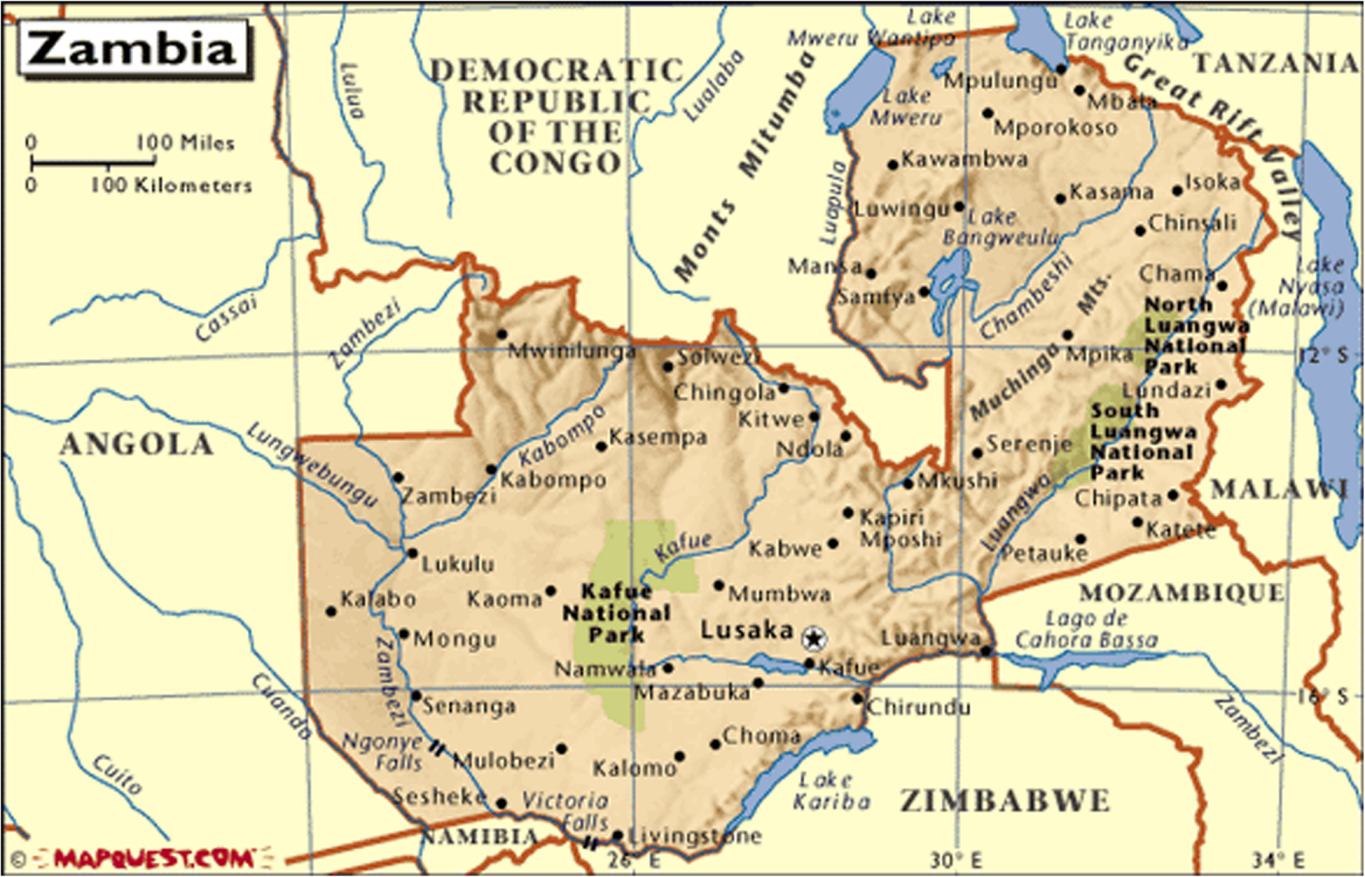
Map of Zambia.

The study was conducted in Lusaka (the capital city of Zambia) and Kapiri Mposhi district, located in the Central Province of Zambia (Figure 1). Lusaka was selected because this is where the strategic team members were based. Furthermore, this is where the MoH Headquarters – the body responsible for coordinating all health policies – is located. The other study area, Kapiri Mposhi district, was chosen because it is one of the rural districts where the CHA strategy is being piloted. Training of CHAs was conducted in Ndola district which is located on the Copperbelt Province, about 100 km north of Kapiri Mposhi.

### The study design

A qualitative case study methodology was used to analyse the CHA development process in Zambia. The case study methodology is an empirical approach that investigates contemporary phenomena within a real-life context, where the boundaries between phenomena and context are not clearly evident and in which multiple sources of evidence are used [28]. The case study approach was considered as appropriate for this study because the CHA strategy was developed within a complex context, which involved social interactions, and which was dependent on multiple local and global influences. This approach enabled us to capture the relations among actors in the process of developing the strategy, as well as the realities of the context in which the strategy was formulated.

The study used purposive sampling to select respondents for the key informant interviews based on their role in developing the CHA strategy and coordinating CHA activities: these included strategic team members, MoH staff, and community members. NGOs working with CHWs were not part of the key informant interviews as they did not participate in developing the CHA strategy and they were not familiar with the strategy at the time of data collection.

### Data collection techniques

#### Key informant interviews

Data collection was conducted from January 2012 to August 2012. Key informant interviews were conducted with members of the CHA strategic team and with senior health officials in Kapiri Mposhi district. Using the list of CHA strategic members, interview appointments were made to strategic team members through visits to the offices while appointments for other staff from the MoH were made through phone calls. Although attempts were made to schedule interviews with all strategic team members, only nine out of fifteen members of the CHA strategic team were interviewed. The other six could not be interviewed as they were unavailable during the study period. Two interviews were conducted with the main supervisors for CHAs in Kapiri Mposhi district. Major themes in the interviews included involvement of stakeholders in the process of developing the strategy and their perspectives on the content of the strategy.

#### Participant observation

Part of the data collection process included attending a workshop on 1^st^ November 2011 at Kingfisher Lodge in Lusaka district whose title was “*Community-based Health Volunteers: Designing Research to Inform Policies and Practices that Increase Effectiveness and Sustainability of this Valuable Health Cadre in Lusaka*”. The aim of the workshop was to inform institutions and organisations working with CHWs in Lusaka about the key issues in the strategy. In attendance were delegates from the MoH and NGOs who work with CHWs. Informal discussions were held with representatives of the NGOs during this workshop and other stakeholders who were part of the initial process of developing the CHA strategy, but who were not part of the final process.

#### Documentary review

Review of documents was another data collection method. All reports, studies and other materials on CHW services in Zambia and other countries used in developing the strategy, were included [29-32].

### Data analysis

All interviews were recorded digitally and later transcribed verbatim. Data analysis was conducted manually and followed a thematic framework analysis approach, which is used to classify and organise data according to key themes, concepts and emergent categories [33]. First, interviews and transcripts were thoroughly read. This was followed by the development of codes and categories, a process that was guided by the elements of the Walt and Gilson policy analysis framework (content, context, actors, and process) [25], as well as the case study approach. This approach was relevant in analysing the complex relationships among actors as well as the context.

Then, the codes and categories were cross-checked with the interview transcripts in order to ensure that they were applied to relevant responses found within and across the interviews. The focus was placed on identifying, summarising, and retaining the patterns and similarities, differences, and new emerging themes. Data were then triangulated with other sources such as the information gathered through informal discussions and review of documents. Although the process is presented as a linear process, it is important to stress that this was an iterative process that involved continuous shifting back and forth from participants’ narratives to the researcher’s interpretation of what the informants meant [34].

### Ethical issues

Ethical clearance to conduct the study was obtained from the University of Zambia Biomedical Research Ethics Committee [IRB 0001131 of IORG 0000774, reference number 009-10-11]. Verbal consent was sought from the key informants in the study. This was preceded by an explanation to the participants regarding the research objectives and aims. Furthermore, informants were notified that they were free to withdraw from the study at any point. Study participants were also assured of confidentiality during and after the study period.

## Results

The policy analysis of the process of developing the Community Health Assistant strategy produced several issues which have been grouped into two broad categories: setting the policy agenda and the policy formulation process. Within these sections, how the interaction among actors as well as the interaction between context and actors shaped the policy process and content have been described.

### Setting the policy agenda

The policy agenda consists of issues that policy makers agree to consider at a given time. Agenda setting is a process through which actors negotiate to have their interests considered by policy makers.

#### The role of actors and context in setting the policy agenda

Analysis of secondary data and interviews indicated that the MoH started thinking of establishing a new group of workers that would help address the huge gap for human resources for health in 2006. The Directorate of Human Resources and Administration at the MoH headquarters played a key role in placing the issue on the policy agenda. The first proposal, drafted in 2007, proposed that the new group of workers be called Assistant Nursing Officers (ANOs). The GHWA, a global initiative for addressing human resources for health crisis launched in 2006, funded development of the proposal.

The GHWA is made up of partners and organizations whose objectives and work programmes are related to or supportive of the health workforce. The Secretariat of the GHWA is located at the WHO headquarters in Geneva. The alliance works with partners to advocate for and catalyse actions to resolve the Human Resources for Health (HRH) crisis at country level. In Zambia, the alliance members are the Zambia UK Health Workforce Alliance, which provides a focal point to help ensure that Zambia’s health needs and requests for support and mutual development are known and understood by UK organizations, and the Zambian International Health Alliance. Zambia’s relationship with the GWHA started in 2006 when the country hosted the sub-regional meeting on HRH, convened by the Technical Working Group of the GHWA [2,23].

When the proposal was presented before stakeholders, which included the General Nursing Council, it was rejected. Stakeholders argued that it was not very clear as to what kind of assistance ANOs would provide. The opposition that they faced the first time the concept was introduced to stakeholders was explained as follows:

“*This first concept failed because we wanted it to be registered by the General Nursing Council*. *We had some problems coming up with what to call these people*. *We first called them Assistant Nursing Officer*, *but we had to change the name*. *Because when we presented it before stakeholders in the Ministry of Health*, *it was rejected.*” (Strategic team member).

The other reason for rejecting the proposal was the argument that the government was facing challenges in improving the conditions of service for the existing health workers, and that creating a new category of workers would strain the government finances further. Having rejected the proposal, stakeholders resolved to channel part of the funds from the GHWA towards training of nurses. The general attitude of the stakeholders, when rejecting the proposal, was explained as follows:

“*Ha*! *You should have seen the reaction from the nurses when I presented the idea to them*. *They were very annoyed*. *They told me that* ‘*Look the government is not paying us enough money and you want to introduce another level of health workers who will need money from the same government*, *no*, *we reject the proposal*’.” (Strategic team member).

Following the rejection of the concept, the pioneers of the strategy decided to revisit it in 2008. The goal was to make it attractive and acceptable for the statutory bodies and health unions. In refining the concept, the MoH sought technical assistance from actors outside the country, and they also referred to CHW-driven programmes in other countries.

Ethiopia, being one of the first countries in Africa to formalise the services of Health Extension Workers (HEWs) in 2005 under the Health Extension Program (HEP), was a major reference point for the MoH in shaping the policy agenda. It was reported that the MoH in Zambia was inspired by evidence which showed that the HEWs had positively contributed towards improved maternal health services at community level in Ethiopia. Therefore, staff from the Directorates of Human Resources and Nursing Services at the MoH headquarters undertook a study tour to Ethiopia to understand how the HEP was working. Other similar study tours were undertaken to Malawi.

“*We undertook a study tour to Ethiopia just to understand how the Ethiopian government had managed to integrate the health extension workers in the Ministry of Health before we started developing the CHA strategy.*” (Strategic team member).

Furthermore, during the conference organised by the GWHA in Uganda in 2008, members from the Directorates of Human Resources and Nursing Services at the MoH headquarters presented the concept. Representatives from the GHWA and other stakeholders advised them to change the title from ANO to Community Health Worker (CHW).

“*We started thinking hard on how we were going to refine the concept*. *Then*, *there was a conference in Uganda and we advocated for developing another cadre of health workers to help the existing workers*. *Donors liked the concept*. *We were advised to change the name from Assistant Nursing Officer to Community Health Worker.*” (Strategic team member).

Based on the advice from the conference, the MoH revised the title from ANO to CHW. After further brain storming, the title was changed from CHW to Community Health Assistant (CHA). This change was done to distinguish this new cadre from the CHWs, since unlike CHWs who are volunteers, CHAs would be placed on the government payroll.

### CHA strategy formulation process

**Figure 2:**
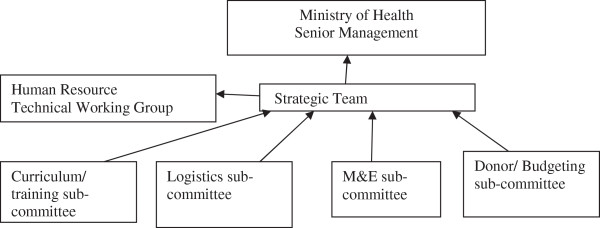
Planning structure for the National CHA strategy.

Having refined the concept, in 2009, the MoH formally appointed a strategic team to facilitate the process of formulating the strategy. Its mandate was to engage stakeholders and regularly update senior management and the human resources technical working group at the MoH on the process. It was also required to collaborate with four sub-committees, namely curriculum, logistics, monitoring and evaluation, and budgeting (Figure 2).

The strategic team was appointed by the Permanent Secretary for the MoH. The team mainly consisted of staff from various directorates/departments under the MoH headquarters. For example, there were representatives from the Directorate of Human Resources and Administration, Public Health and Research, and Technical Support Services as well as Nursing Services. Others were from the Planning, Health Promotion, and Child Health Services departments. Only two members were drawn from the MoH directorate which is not under the MoH headquarters, the National Malaria Council Centre. The strategic team was chaired by the Directorate of Human Resources and Administration.

Once the strategic team was in place, the MoH conducted a situational analysis with 18 implementing partners, 76 CHWs and the District Medical Officers (DMOs) to understand the roles, scope and challenges of CHW-driven programmes. The GHWA funded the situation analysis. However, it was not clear who from the MoH coordinated the analysis and the partners that were interviewed, or how respondents were selected.

The situational analysis showed that DMOs recommended standardisation of recruitment, education requirements for recruitment, training and supervision of CHAs, and remuneration, with monetary incentives being the most preferred type of incentive by the DMOs and CHWs. Implementing partners recommended standardisation of training, remuneration and supervision for CHAs. All respondents unanimously supported the need for providing periodical refresher courses to CHAs. However, implementing partners were silent on the type of remuneration to give to CHAs or on the educational requirements for recruitment. Further, only about half of them supported standardisation of recruitment processes for CHAs [19].

Having concluded the analysis, the MoH organised a consultative meeting to negotiate with unions and professional health bodies on the need to develop a new group of health workers in Zambia. Staff in the strategic team from the Directorate of Human Resources with the assistance of those from the Directorate of Nursing Services at the MoH headquarters chaired the consultative meeting. Although it was termed consultative, the meeting was in practice strategically organised to enable the pioneers of the CHA strategy to push the concept through.

To convince the unions and nursing bodies on the relevance of developing the cadre, the strategic team members reported that, apart from revising the title, they also had to change the emphasis regarding the area of operation for CHAs when presenting the concept to the stakeholders. They stressed that CHAs would be based in rural areas, the areas where the pioneers of the strategy knew that most professional workers would not want to work from. This is how one of them explained how they managed to convince the stakeholders:

“*We did our homework before presenting the idea to them*. *For this one*, *we presented the strategy like it was going to be purely in the village*. *We asked them if they were willing to go and work in the village*. *They all said no*. *Then we reminded them that most of their grandmothers were in the villages*. *So we asked them* “*who should provide health services to them*?” *They all responded* “*Another cadre*! … *another cadre*!” *So we told them that the same cadre is the one we were now calling Community Heath Assistants.*” (Strategic team member).

#### Limited consultation and consideration of divergent views

There were mixed views regarding the process of formulating the strategy. While some strategic team members were satisfied with the process, others were not. Those who were dissatisfied reported that apart from being insufficiently consultative, the process was also rushed. It was reported that coordinators of the process did not adequately consult on whether or not to launch the strategy, or when to launch it. Furthermore, some of the members of the strategic team complained that they were not requested to review or comment on the strategy before it was launched. Two members stated that they had thought that they were still at a brainstorming stage and were surprised to learn that the strategy had already been finalised and launched. The problem with regard to the pace of formulating the strategy was explained as follows:

“*It was not done properly*. *It was rushed*… *Because as far as I*’*m concerned*, *we were still at the brain storming stage and then we only heard later on that CHAs had been recruited and trained*… *The idea went too fast.*” (Strategic team member).

It was further suggested that the process was less tolerant towards stakeholders with opposing views. Two people, who had been part of the CHA strategic team in the beginning, reported that they were side-lined from participating in subsequent activities because they had opposed the idea of developing the strategy when it was first introduced. These people explained that they had argued that it was better to concentrate on addressing problems that nurses were experiencing rather than to focus on creating a new group of workers whose scope of work would be less than that of nurses.

#### Poor communication and feedback processes

Several members of the strategic team were not happy with communication processes. The members complained that there were no regular updates on what was being done, by whom, and at what time. The existence of poor communication systems within the strategic team could also be noticed with regard to accessibility to the strategy and awareness of its contents. Several members stated that they had not seen the developing document at the time of this study. Meanwhile, even for those who had seen it, fewer than half had read through the strategy. Because of this, not all members were fully aware of some key themes contained in the strategy. For example, although the monthly incentive figure for CHAs during the pilot phase was clearly indicated in the strategy, most respondents, including those from the strategic team, were not aware of it. Some of them stated that they could not remember agreeing on the salary scale for CHAs.

“*We failed to agree on the issue of remuneration*… *I mean we could not agree on how much to pay them.*” (Strategic team member).

Apart from having insufficient knowledge on some of key issues, the other feature of poor feedback processes was about members having contradictory information on some key issues in the strategy. For example, while several respondents stated that CHAs would be based within the community, one member informed that the MoH would construct houses for CHAs to live in at the health posts.

#### Donors and politicians influential but invisible actors

Review of data further showed that apart from influencing the setting of the agenda, several institutions and organisations also supported the process of formulating the strategy and piloting it by offering financial and technical support. The main sponsor of the CHA strategy was the Department for International Development. Other funders included the Zambia Integrated Health Systems Strengthening Program, a United States Agency for International Development program designed to increase utilization of health services through a health systems strengthening approach, which financed salaries for the lecturers. Technical support was provided by the Clinton Health Access Initiative, while the United Nations International Children’s Emergency Fund contributed study materials to the training school.

Donors and politicians also actively contributed towards shaping the process by constantly urging the strategic team to quickly finalise and implement the strategy. For example the GHWA, reportedly used to phone the former Minister of Health asking him why it was taking too long to implement the concept, and that the Minister would then urge the committee to speed up the process. Review of documents also showed that the GHWA had recommended, in March 2010 (before the launch of the strategy in August 2010), that the strategy be implemented quickly. It also suggested that the content of the policy be similar to that of the HEW’s policy in Ethiopia.

“*The Community Health Worker National Strategy of 2009 lays out options for a clear and effective CHW strategy*, *policy and structure*. *This strategy should be implemented as soon as possible*. *More CHWs must be trained in order to successfully implement this strategy*, *and Zambia would do well to consider a policy similar to the one in Ethiopia*, *in which they are trained over a period of six months*” [23], p. 1].

Further analysis of the strategy also showed that it was in line with resolutions of the consultative meeting organised by the GHWA in 2010, in Switzerland. This meeting recommended that countries should consider integrating CHWs fully into the health system or national human resources plan [13], in order to strengthen primary health care.

#### Engagement of civil society and CHWs

Interviews with the strategic team members suggested that the involvement of civil society and CHWs in the process of formulating the strategy was not very clear. Furthermore, no CHWs and civil society reviewed the strategy before it was launched.

Regarding the unclear involvement of civil society and CHWs in the process of formulating the strategy, informants from the Human Resources Department at the MoH explained that some stakeholders could not have been fully involved because the work was done on a voluntary basis. They further suspected that others could have failed to attend meetings due to commitments and ineffective communication systems.

“*We used to call for meetings but people did not just turn up*. *Maybe because they were busy or they did not receive our invitation or they wanted to be paid allowances*. *But this work was done on voluntary basis.*” (Strategic team member).

#### Differential notions of policy content

Analysis of interviews and the strategy showed that, although positive issues were identified within the policy content, there were also some limitations. Some of the policy gaps were attributed to policy process shortcomings: less consultative, poor communication, and an excessively speedy process. Positive policy attributes included standardisation of the training programme for CHAs. The training programme was not only longer than that for CHWs, but it was also more comprehensive as it covered several primary health care topics. Furthermore, the programme was developed by the key training institutions in Zambia which included the General Nursing Council, Health Professional Council of Zambia, Chainama College of Health Sciences, Dental School, University of Zambia School of Medicine, and Lusaka School of Nursing.

Controversial policy contents included the plan to pay CHAs monthly salaries. It was argued that this plan had the potential to destroy the culture of volunteerism which has long been part of the working culture of the community-based health workforce in Zambia. They stated that although the new cadre would be called CHA, the fact that they would be based in their communities had the potential to discourage some CHWs from effectively performing their tasks, as they may also want to be paid salaries. Since there are more CHWs than there will be CHAs, it was feared that any possible conflicts between these two groups could retard the progress that some of the health programmes that depend on CHWs’ services had secured. One key informant explained this dilemma as follows:

“*We have thousands of CHWs in the field doing good work and who are working on voluntary basis*. *It is part of community spirit to volunteer*. *Now*, *can you imagine*, *you pick a few people from the same community*, *train them*, *and then send them back to community*, *and start paying them monthly salaries*. *It is a disaster.*” (Strategic team member).

Analysis of CHA strategy and interviews also showed that the policy did not provide clear plans for professional development and refresher courses for CHAs despite being recommended in the situational analysis. The other concern was about the lack of a clear plan on how to address staff attrition once the target of about 5,200 CHAs was attained in the final phase of the programme. Most respondents noted that, since CHAs would be more skilled than CHWs, it was highly likely that they would seek other employment opportunities in the future, thereby reducing the number of CHAs in the community.

## Discussion

This paper has analysed the process of developing the CHA strategy in Zambia by trying to understand the policy context and stakeholder involvement, and how these shaped the policy process and content. The study has provided insight into the evolution of the design of a policy framework aimed at integrating CHWs into the health system. It has tried to show how power differences among actors influence who participates, how they participate, the direction of the policy process, as well as its content.

### Actor involvement in setting the policy agenda

The findings of this study show that the policy development process was driven by the need to address the human resources for health shortage in the country. Our analysis also reveals, however, that it was just a few actors from the MoH headquarters who worked to place the issue on the policy agenda and who participated in formulating the strategy. Agenda setting, which is a phase when issues get selected and prioritised to come into the policy agenda [35,36], was mainly informed by the Department of Human Resources and Administration from the MoH. Actors from this department took up the role of “policy entrepreneurs” or pioneers of the idea of developing a new group of workers [36].

The study showed that agenda setting may not always be a straightforward process, as some stakeholders may be sceptical of new ideas. Scepticism may result in delays in confirming agenda items [36,37]. For example, when developing the HEP in Ethiopia, which is one of the first programmes in Africa to formalise HEW services, not all partners in Ethiopia supported the initiative as they feared that HEP would not positively contribute towards maternal health [38]. Similarly, the health professional bodies in Zambia did not support development of the CHA strategy at first as they did not understand the concept and were pessimistic about its potential benefits. The concept was only placed on the agenda through several negotiations and consultations between the pioneers of the concept and health professional bodies. Negotiations and consultations are an important part of agenda setting as they facilitate refining of the policy issue by “softening up” stakeholders initially opposed to the issue, and by expansion of the policy issue within or beyond the local context and actors [36].

International actors who participated in the process and who subsequently contributed towards refining the concept to an agenda item included the GWHA. The influence of international actors and global context on national policies in LMICs has been widely documented [39], with international bodies potentially playing the role of indirect actors in national policy reform. These actors may not only influence agenda setting but also the process and content. For example, documentation on the performance of HEWs in Ethiopia partly motivated the MoH to develop the CHA programme [19,40]. The other research evidence used in developing the strategy was from Malawi. The only data from the Zambian context was from the National Situation Analysis which mainly focused on gaps in human resources for health and challenges faced by CHWs [19]. Furthermore, the GHWA also recommended that Zambia should develop a strategy similar to the HEP in Ethiopia and that it should be implemented quickly [23].

Review of the strategy showed that its content also reflected some of the key messages from the Global Consultation on Community Health Workers organised by the GHWA in 2010. These included the recommendation that countries should consider integrating CHWs into national health workforce plans, as well as the need to ensure a regular and sustainable remuneration stipend for CHWs [13]. This consideration of the views from global actors in developing the policy, by interest groups wishing to influence the policy-making process, could be viewed as one strategy of securing a recognised position with the donors/funders [41], which may in turn guarantee financial and technical support from the agencies during the policy implementation.

### Power imbalance among actors in CHA formulation process

Although central agencies and policy entrepreneurs could be useful in policy making, literature suggests that their involvement may limit participation of local stakeholders in policy formulation [5,42]. Limited stakeholder involvement could work against the ideal policy making process which should accommodate relevant government bodies, civil society, community structures, private-not-for-profit, and health professional groups in health policy reform [13]. While stakeholder consultation is recommended, the findings of the study suggest that consultation may in practice be deployed strategically in order to advance the interests of key drivers of the policy, or donor conditionalities. Strategic consultations could include the reported side-lining of those with opposing views from participating in the CHA formulation process, and the inclusion of contentious issues in the CHA strategy without involvement of several strategic team members.

Our findings therefore support other studies on health policy reforms that argue that actors and interest groups exercise different degrees of power in influencing the course of the health policy reform process [5,42-44]. The degree to which actors are able to influence the course of policy making process as well as the content of the policy depends, amongst others, on their perceived or actual power [25,27]. This power may be exercised by making sure that issues that reflect the values and beliefs of those in top management, or of the pioneers of the idea, reach the policy agenda. It may also seek to ensure that opposing views in health reforms are not given a platform [5,45].

In addition, the negotiations and compromises with nursing and statutory bodies highlight the political nature of the policy process. Our findings resonate with the view that “*policy change is not simply a technocratic process based on rational analysis, and that knowledge alone is not sufficient for policy change. Policy is profoundly political*” [45], p. 8]. This means that policy is set by the various ways in which people exert control, influence, or power over each other [42]. Perhaps a more fundamental problem relates to the fact that, although the importance of politics in policy reform has been acknowledged, there is also broad agreement that political issues are rarely analysed by the people involved at all stages of the process, and particularly with respect to the interactions between international donor agencies and recipient country governments or institutions responsible for policy reforms in LMICs [43,46]. One possible explanation for this insufficient political analysis could be that although many actors may have their own clear political agendas which they want to pursue in relation to the policy, these often remain unstated, and therefore invisible to other actors. This is likely to keep particular political issues out of the official rhetoric associated with the policy.

The lack of involvement of CHWs in formulating, finalising, and launching the strategy showed the lack of voice and power of the community-based health workforce in the contemporary policy development process and reform [5]. This insufficient involvement may not be unique to this study, as it has been documented that public participation in deciding the policy agenda in LMICs has often been limited [43]. Perhaps this limited involvement could be explained by the political will or technocratic model which assumes that decisions by senior managers or leaders or a reform champion are necessary and sufficient for policy change and that these leaders are rational actors maximizing the public interest [47], which, in the context of this study, could include addressing the chronic human resources for health gap in Zambia. Based on the findings of the study, one may argue that as desirable as engaging multiple stakeholders may be, achieving broad consultation in formulating policy may not be feasible in situations where it is difficult for stakeholders to agree on key policy contents due to different interests, or where key policy developers may have specific issues to advance.

Advancing the views of a limited group of stakeholders in the policy formulation process has the potential of producing a policy that does not reflect the interests of the majority of stakeholders, or consider key issues within the given context. For example, although it has been widely documented that movement of health workers from one job to another is a major problem affecting human resources for health globally [2], the CHA strategy did not comprehensively consider how to address staff attrition or professional development of CHAs. This policy gap could limit the possibility of building a sustained and effective strategy as a sustained CHW strategy requires viewing the CHW position not as an end-point but as a means to an end, and accepting a high degree of turnover which in turn requires putting in place measures to address the anticipated turn over [15]. Furthermore, although several studies have shown that acquired skills are quickly lost, and therefore refresher training is of crucial importance for sustaining the quality of performance and sustainability of CHWs [1,48], the CHA strategy appears not to have adequately considered general professional development and progression matters. This limited consideration of vital issues in the strategy may resonate with Walt and Gilson’s view that much health policy wrongly focuses on content of reform and neglects the context within which policy is developed [26].

Apart from contributing to policy gaps, limited participation could potentially affect implementation of the CHA strategy, as collaborative planning with all relevant stakeholders at the initiation of the design process of any CHW programme, whether for HIV or other service delivery, is a key factor for the success of the programme’s development as well as its implementation [9]. Limited stakeholder involvement could result in weak collaborative systems and referral networks between CHAs and other health service providers at community level. For example, studies conducted on the HEP in Ethiopia showed that poor referral systems affected the work performance of health extension workers [49,50]. It has therefore been suggested that for community-based workforce to work effectively, apart from formalising their services, there is a need to create an appropriate working environment and conditions within the health sector. It has been argued that while the renewed attention to CHWs is welcome, scaling-up of CHW programmes without considering ways of simultaneously strengthening health systems may comprise the quality of the CHW services [48].

### Strengths and limitations of the study

Trustworthiness of the research findings was enhanced by triangulating data obtained from the key informant interviews with those obtained from documentary review and informal discussions. Although most of the information was similar, some disagreements were observed between interviews and documentary review regarding placement and remuneration for CHAs. Furthermore, trustworthiness was achieved by compiling, documenting, and including all data during the analysis process.

The study was unable to include all the relevant actors. Although efforts were made to interview all the 15 CHA strategic team members, only 9 were interviewed as others were not available during the study period. NGOs working with CHWs were not included in the study because they were not part of the process of developing the CHA strategy. Lack of inclusion of these stakeholders is a limitation as they could have given some important perspectives of the process. Despite these limitations, the rich description of the policy process in this study provides a valuable contribution to the knowledge base on policy reforms for community-based health workforce in LMICs.

## Conclusions

The CHA policy making process was found to be not only complex but highly political, suggesting that the policy making process was accompanied by power imbalances. It seems reasonable that stakeholders should not be naïve in assuming that they can fully control the process or otherwise provide substantive input that is agreed and acted upon without an active iterative advocacy process built on context needs and demands. It is important to recognise that, in spite of the democratic rhetoric that the process may seem to suggest, the practice may be different, and this could have critical strategic and sustainability implications. For example, the frequency and type of feedback on the progress of the policy process may not be the same to all stakeholders. Furthermore, influencing the policy process may also be contingent on one’s position in the political hierarchy far more than one’s knowledge and understanding of the issue. We have sought in this paper to demonstrate that, because of power imbalances among stakeholders, the policy process may focus on the content and neglect to include all the relevant actors and contextual factors. This may have serious policy implications as those actors who may not be in support of such issues or may not be happy with the general policy development process may not support or participate in policy implementation, monitoring and evaluation.

The study provides insights not only to the MoH in Zambia, as it pilots and prepares to roll out the CHA strategy, but also to other countries that engage CHWs in health service provision. Furthermore, the study contributes towards the existing knowledge about reforms on community-based health workforce by comprehensively discussing some of the dynamics that shape health policy reform. This could help stakeholders, researchers and policy makers to adequately interpret and effectively guide implementation, monitoring and evaluation of similar health policy reforms.

## Abbreviations

ANO: Assistant nursing officer; CCF: Country coordination and facilitation; CHA: Community health assistant; CHW: Community health worker; DMO: District medical officer; GHWA: Global health workforce alliance; HEP: Health extension programme; HEW: Health extension workers; LMICs: Low and middle income countries; MoH: Ministry of health; WHO: World Health Organization.

## Competing interests

The authors declare that they have no competing interests.

## Authors’ contributions

All four authors contributed towards the study design. JMZ carried out the data collection. JMZ, JK, CM and AKH analysed the data. JMZ drafted the manuscript and all authors contributed towards revision of the manuscript. All authors read and approved the final manuscript.
